# Supplementary material for: Seminoma and Embryonal Carcinoma Footprints Identified by Analysis of Integrated Genome-Wide Epigenetic and Expression Profiles of Germ Cell Cancer Cell Lines
Source: PLoS One. 2014 Jun 2;9(6):e98330. doi: 10.1371/journal.pone.0098330 (PMC4041891; doi:10.1371/journal.pone.0098330)
Supplement: File S1 — ZIP file containing DMRforPairs output for significant regions. Please start from the html files. (ZIP) [file pone.0098330.s008.zip › figures/25833.pdf]

RegionID: 25833, chr20:61051317−61052060−M_values

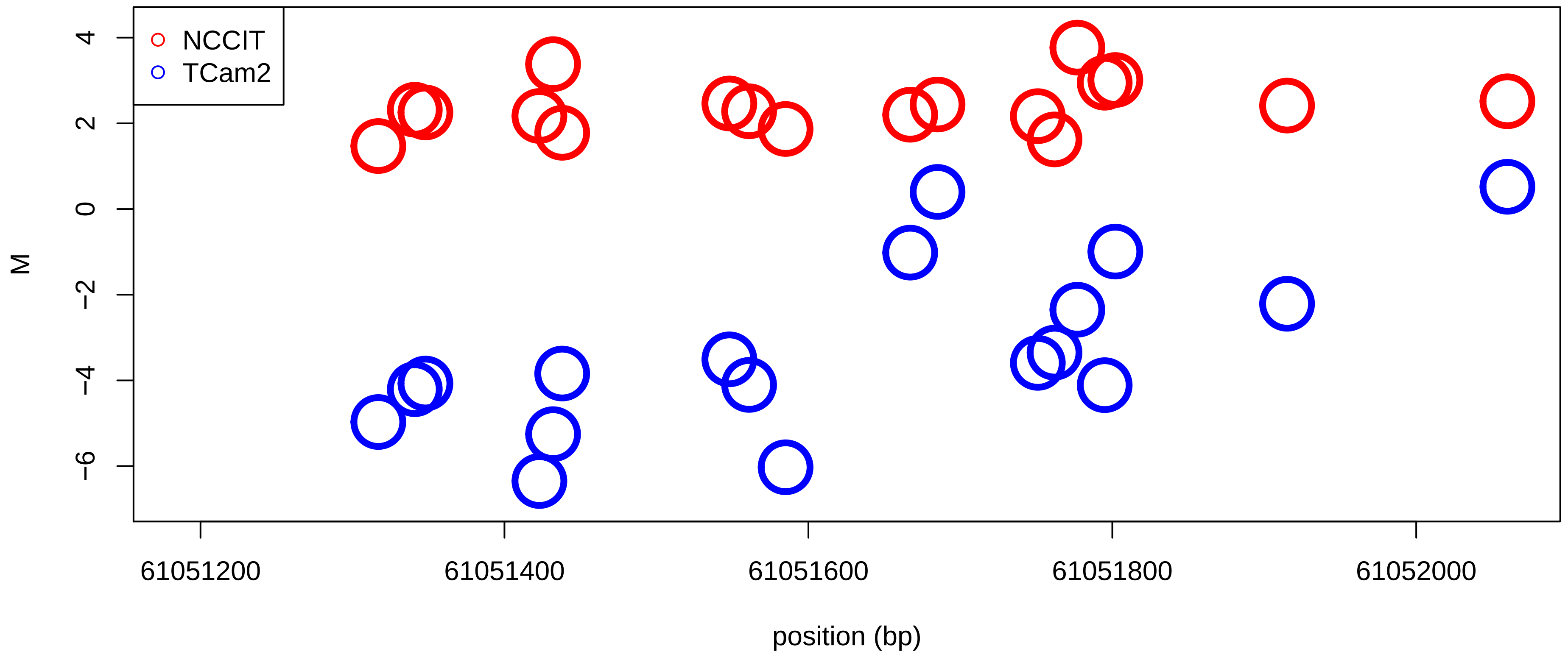

RegionID: 25833, chr20:61051317−61052060−Beta_values

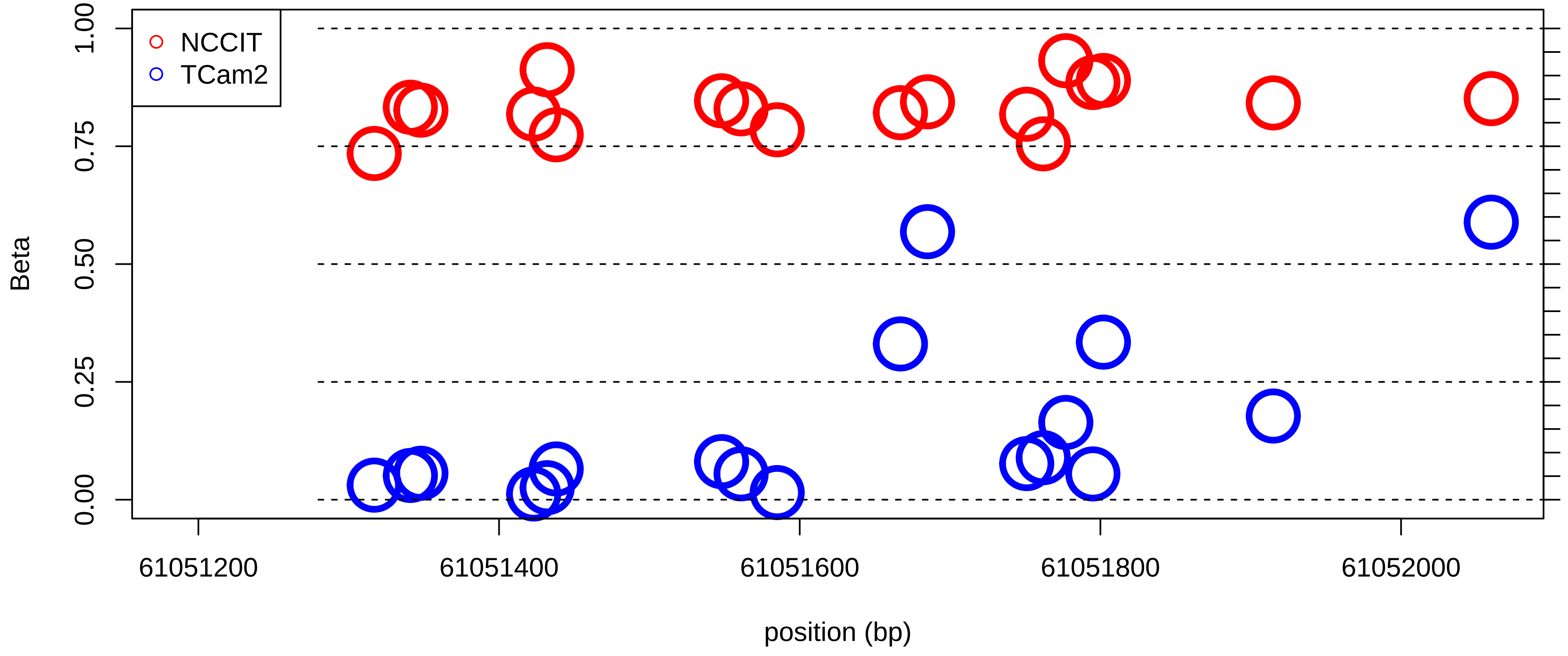

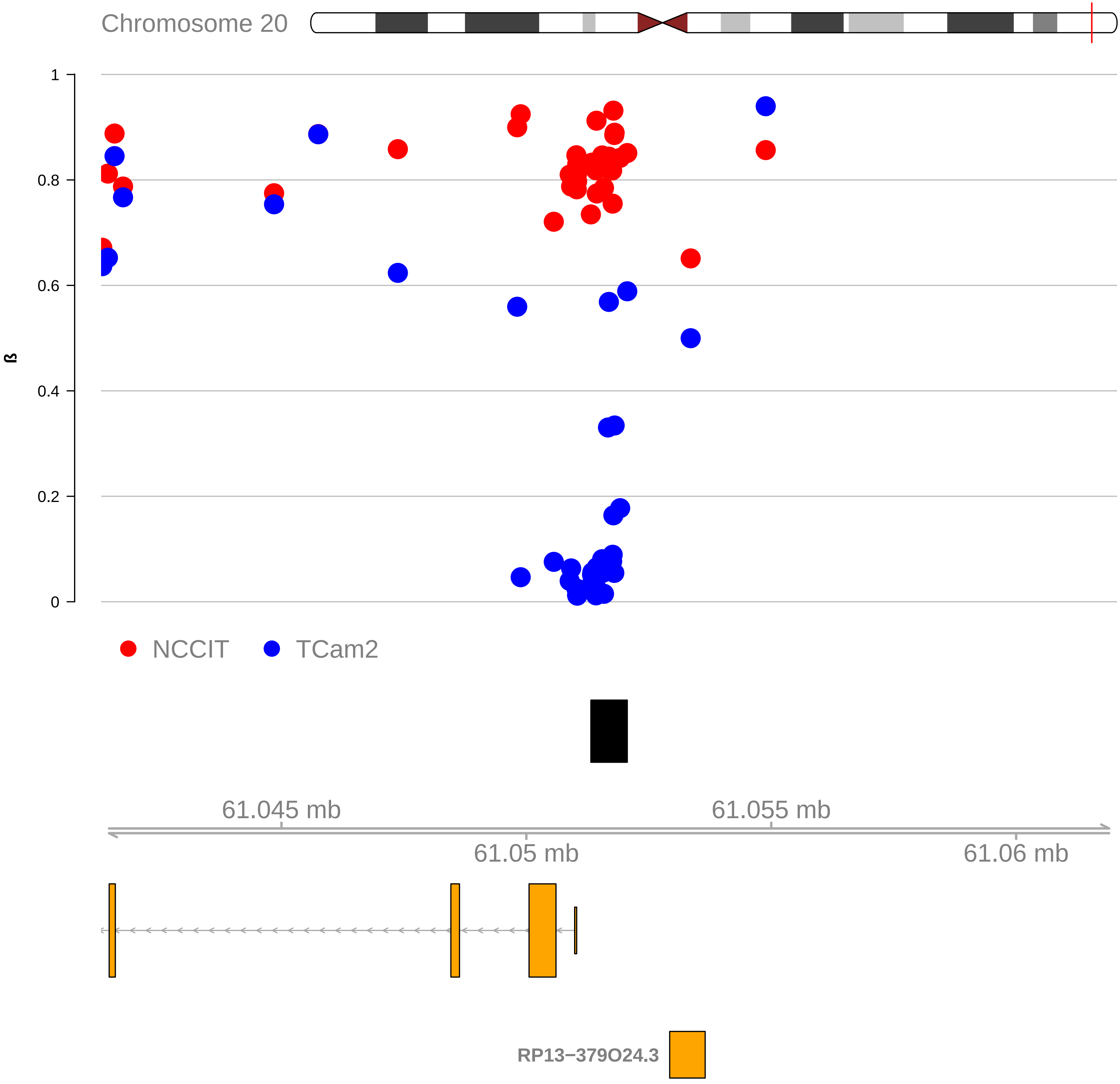

Chromosome 20
1
0.8
0.6
0.4
0.2
0
ß
NCCIT
TCam2
61.045 mb
61.055 mb
61.05 mb
61.06 mb
RP13−379O24.3
